# Vaccine acceptance in rural India: Engaging faith leaders as vaccine ambassadors

**DOI:** 10.3389/fpubh.2022.979424

**Published:** 2022-09-20

**Authors:** Preetika Banerjee, Rajeev Seth, Baldeep K. Dhaliwal, Alexis Sullivan, Yawar Qiayum, Betty Thankachen, Svea Closser, Anita Shet

**Affiliations:** ^1^International Vaccine Access Center, Johns Hopkins Bloomberg School of Public Health, Baltimore, MD, United States; ^2^Bal Umang Drishya Sanstha, New Delhi, India

**Keywords:** vaccine acceptance, vaccine hesitancy, faith leader influence, health promotion, India, health communication

## Abstract

**Introduction:**

Religious faith is a key marker of identity and shapes community perspectives and trust. Faith leader involvement in vaccine campaigns in India have been beneficial to counter misinformation regarding infectious diseases such as polio. Faith leaders are influential stakeholders who bear potential to enhance public confidence in vaccine campaigns.

**Context:**

While vaccine coverage has been increasing in India, inequities abound, especially in populations with historically low vaccine confidence. The COVID-19 pandemic has led to major disruptions in delivery of routine immunization services for children. To address these challenges, we co-designed interventions aimed at contextual communication strategies and peer support. Engaging faith leaders was an important part of this intervention. In this report we describe our experience and highlight the perspectives of faith leaders and their expectations of the outcomes for this intervention.

**Programmatic elements:**

The CIVIC Project, conducted from January to December 2021 aimed to engage caregivers, community health workers and key stakeholders, particularly, faith leaders in co-designing interventions to address vaccine hesitancy in Mewat. The project, deeply rooted in community based participatory research, used a three-E approach (Exploration of community perspectives, Establishment of vaccine trust and awareness, Engagement in vaccine promotion activities) to successfully engage faith leaders in the design and dissemination of media messages advocating for vaccine acceptance and uptake.

**Lessons learned:**

The involvement of faith leaders in the intervention benefited the community in two ways. First, faith leaders were spotlighted *via* videos, often disseminating advice and personal anecdotes about vaccines, thus reassuring caregivers and community members who previously expressed distrust in vaccines. Second, involvement of trusted faith leaders provided a platform for a two-way dialogue for the community to openly discuss and address myths and misconceptions regarding vaccines. This project provided the learning that co-creating interventions with faith leaders who are often gatekeepers of close-knit communities can lead to the development of vaccine positive messaging that community members relate with, motivating increased vaccine confidence.

## Introduction: The role of faith and faith leaders in health promotion programs

Religious faith represents a key social and environmental driver that can influence an individual's beliefs, their health behavior, and practices (1). Health promotion programs rooted in the involvement of faith-based organizations and in the engagement of faith leaders as advocates for disease prevention and treatment have proven effective in bringing about positive change in community health (1).

In 2019, the World Health Organization (WHO) identified vaccine hesitancy among the 10 threats to global health (2). Vaccine hesitancy has become inextricably linked to religious faith in several ethnic communities across the globe. Concerns about vaccine safety combined with circulation of misinformation poses severe threats to public health and attainment of herd immunity against infectious diseases (3). Addressing vaccine hesitancy through multi sectoral partnerships and facilitating faith-based dialogues is imperative to drive acceptance and uptake of COVID-19 vaccines (4).

Historically, communication strategies centered around faith leaders have led to favorable outcomes in tackling misinformation and reducing the spread of infectious diseases, including poliomyelitis (polio), human immunodeficiency virus (HIV), and Ebola (5). Evidence from polio eradication efforts in India and Pakistan shows that minority Muslim communities were among the hardest to reach. Low polio vaccine uptake was linked to vaccine hesitancy and misinformation among community members. In these settings, participation of faith leaders in polio campaigns enhanced public confidence and bolstered the credibility of the Polio Eradication Initiative leading to an increase in vaccine coverage (5).

In India, the UNICEF-managed Social Mobilization Network (SmNet) specifically countered religious vaccine refusals by approaching faith leaders to speak about the impact of polio during Friday prayers and regularly make mosque announcements (6). Building off the successful elimination of polio in India, the involvement of faith-based leaders and grassroots-level involvement in immunization efforts was expanded to promote routine immunization services, sanitation, and other maternal and child health programs in India (7, 8).

## Context: Addressing gaps and challenges for vaccine acceptance in Mewat, Haryana

India is currently tackling pockets of populations unwilling to receive or unaware of COVID-19 vaccines (9). Additionally, the COVID-19 pandemic caused major disruptions in routine immunization services for children in India leading to significant declines in coverage rates, particularly during lockdown periods. This left high numbers of children susceptible to vaccine-preventable diseases (10, 11).

In the Mewat district of Haryana State in India, low rates of vaccine coverage have been a long-standing obstacle to health improvement. According to data from the National Family Health Survey (NFHS-5, 2019), 53.8% of children between the ages of 12–23 months were reported to be fully vaccinated with bacille Calmette-Guerin (BCG), measles, and three doses each of polio and diphtheria, pertussis, and tetanus (DPT) vaccines (12, 13). This value lags far behind the national average of 76.4% (14) and falls short of the WHO goal of reaching 80% coverage for all vaccines in national programs for every district as per the Global Vaccine Action Plan (15).

Previous research exploring community perceptions of vaccination in Mewat has highlighted several factors that limit vaccine acceptance, such as fear of side effects, low education levels, poor communication with CHWs and religious factors (16). To increase vaccine uptake and address barriers to vaccination, it is essential to gain a deeper understanding of nuanced community-level factors driving vaccine hesitancy, along with identifying trusted sources for decision-making (16, 17). Faith leaders are often considered one such trusted source in the community, as they influence health behavior across individual and socio-cultural levels (18). Faith leaders are members of the community who are recognized for their role in guiding and inspiring religious and faith-based beliefs and practices of the community, playing an authoritative and influential leadership role.

To meet the challenge of low vaccine uptake in this community, we revitalized social mobilization approaches involving faith leaders. This report highlights findings from the Community Health Worker-Led Intervention for Vaccine Information and Confidence (CIVIC) Project in a rural setting in India. Utilizing a combination of broadcast, print, and social media sources to disseminate vaccine messaging from faith leaders led to improvements in community-level vaccine awareness and acceptance (19). As faith leaders represent a key resource in building vaccine confidence in the community, we explored their perspectives regarding vaccines and engaged them to partner with local health workers in promoting messages of best practices with regard to vaccines.

## Methods

The CIVIC Project was conducted from January to December 2021. The project was designed using a community based participatory research approach, seeking active collaboration from the community members in Mewat in the intervention design. This study was approved by the Johns Hopkins Bloomberg School of Public Health Institutional Review Board (IRB) under IRB submission number 14670/CR717. The IRB determined that proposed activities were exempt under category 2. Under this exemption, the inclusion of participants in our study is identifier recorded and not sensitive. This implies that any disclosure of the human subjects' responses outside the research would not reasonably place the subjects at risk of criminal or civil liability or be damaging to the subjects' financial standing, employability, educational advancement, or reputation.

The interventions for the project were co-designed by the research team and community iteratively. This strategy was used to strengthen the decision-making power of community members, learn from their problem-solving skills and to ensure that the interventions would be tailored to the specific needs of the population.

The intervention with regard to faith leaders consisted of a three-E approach:

1. Exploration of community perspectives
2. Establishment of vaccine trust
3. Engagement in vaccine promotion activities.

### Exploration of community perspectives

The project was initiated with a series of formative interviews (consisting of open and close ended questions) conducted with 10 community members and 10 community health workers (CHWs). Community members recruited for the interviews were involved in a variety of occupations including teaching, social work, farming, and involvement in the panchayat (village council). These formative interviews were followed by two virtual human centered design (HCD) workshops conducted with both these groups (community members and CHWs). These formative interviews and HCD workshops were aimed at understanding community perspectives regarding vaccines and identifying specific barriers to vaccine acceptance that could be addressed through the project (20). The interviews and HCD workshops were facilitated by data collectors who had experience with qualitative data collection. Data collectors were provided with additional training on specific qualitative techniques such as probing and open-ended questions.

Two independent readers conducted rapid qualitative evaluation and thematic analysis on interview responses and documentation from the HCD workshops. The preliminary data from these interviews and HCD workshops overwhelmingly indicated that faith leaders were one of the most trusted sources of information in the community. This suggested that their involvement in multiple aspects of the intervention would be pivotal for improving vaccine uptake and acceptance in the community.

To facilitate faith leaders' involvement in the intervention, the research team approached 10 faith leaders in the area. Through the research teams prior work in Mewat, these faith leaders were identified through purposive sampling. Our sampling strategy enabled us to identify faith leaders who had an established reach and spanning across diverse sections of the community who were well-positioned to provide insights on their individual and broader community perspectives about vaccines. Initial conversations revealed that the faith leaders themselves had several doubts and questions about vaccines due to gaps in their own awareness about the diseases prevented by vaccination and the overall health benefits of vaccines. Until these were addressed, they were hesitant to promote vaccines among their communities.

### Establishment of vaccine trust and awareness among faith leaders

To address faith leader concerns regarding vaccines and boost their confidence with advocating for their uptake in the community, the research team facilitated a series of discussions and feedback with the faith leaders spanning a 6-month period (January–July 2021). The two approaches used to connect with the faith leaders and build their trust were knowledge-sharing and enabling interactions with healthcare providers.

#### Informal knowledge sharing sessions

The research team frequently met with faith leaders to provide them with information about the project and establish a sense of comfort and familiarity between health providers and the faith leaders. During these meetings, the research team encouraged the faith leaders to share their doubts and questions about vaccines. These sessions were planned to be small in numbers (2–3 faith leaders at a time) and informal. Further, the research team made a deliberate effort to be receptive to the faith leaders' questions.

These sessions provided the faith leaders with an opportunity to share their doubts in a safe environment, ensured their concerns were appropriately addressed, and facilitated trust across multiple levels of the community. Maintaining continuous dialogue with faith leaders led to the gradual establishment of trust and dispelling of rumors.

#### Interactions between healthcare workers and faith leaders

While the informal sessions reassured the faith leaders of the positive impact of vaccines on child health outcomes and long-term community health, they were unclear about the issues related to vaccine delivery and unsure of how their involvement could benefit vaccine acceptance. Therefore, the research team facilitated interactions between faith leaders, CHWs and other health professionals, including representatives from polio campaigns.

Community health workers are often women who are from communities they serve in and perform a range of tasks for health promotion and prevention (21, 22). As they have been responsible for sustaining routine immunization services during the pandemic in addition to supporting COVID-19 vaccine drives, CHWs provided insight to the faith leaders on their struggles with combating multiple sources of vaccine hesitancy in their community. Additionally, evidence shows that faith leaders played a major role in polio eradication in India through countering faith-based hesitancy by enhancing community engagement, especially among hesitant populations in Uttar Pradesh, a neighboring state to Haryana (6). Interactions with representatives from the polio campaigns enabled the faith leaders to learn more about these success stories, making them keen to actively participate in the CIVIC project.

The combined approaches of informal knowledge sharing with the research team and interactions with healthcare workers provided the faith leaders with a comprehensive understanding of the benefits that increased vaccine acceptance and uptake would offer to the community. The informal sessions were critical in enabling the faith leaders to comfortably share their concerns. This was complemented by the interactions with the health care workers through which the faith leaders gained knowledge about vaccines delivery and the challenges faced by the healthcare workers during their interactions with the community.

### Engagement in vaccine promotion activities

The interactive sessions for the faith leaders with the research team, CHWs and other health workers set up a strong rapport between the faith leaders and the research term. Collaboratively, they designed activities that would provide platforms for the faith leaders to provide information about vaccines to the community in a manner that would ensure reach to the community despite social distancing norms and in the absence of community gatherings. The two key activities included use of messaging in popular media, and participation in the Community Accountability Board (CAB). Faith leaders were not offered any financial or other incentives to participate in these activities. They co-designed and came forth to volunteer their time and participation in these activities because of their gradually established conviction of the benefits that positive vaccine messaging would offer to their communities.

#### Media messages

Faith leaders, along with other trusted community members (teachers, social workers) spoke in short video messages in which they advocated for vaccines. In these videos, faith leaders spoke about the importance of vaccinating children and shared potential negative long-term impacts of children remaining unvaccinated. These context-specific videos were tailored to the needs of the community and used local vernacular to ensure their message was heard and understood. These short videos were leveraged by CHWs in door-to-door communications with caregivers, and they were shared extensively across social media platforms in Mewat. Of these platforms, WhatsApp was a particularly popular mode of circulating this video. The messages from these videos were also discussed over broadcasting media channels such as the local station Radio Mewat which has been an important awareness building platform for this community for several decades (23). Further, print articles highlighting these messages and the CIVIC project, were published in five local news outlets spanning English, Hindi and Urdu language papers. This multi-pronged dissemination enhanced the reach of messages supporting the value of vaccines for community health.

#### Involvement in community accountability board (CAB)

Observing the positive impact of the videos, the faith leaders agreed to be members of the CIVIC Project's Community Accountability Board (CAB). This CAB of influential community leaders met virtually *via* video conferencing platforms every month to discuss progress of the CIVIC Project, make changes to the project's intervention aspects as needed by the community, and discuss other health issues in Mewat. Given the instrumental role of their endorsement and messages in shaping community perspectives, the faith leaders' decision to join the CIVIC Project's CAB was monumental. The CAB consisted of 10 members, with one Hindu faith leader and two Muslim faith leaders (Maulanas). Through engagement in monthly meetings, the CAB offered a platform for the faith leaders to continue to partake in regular discussions and decisions regarding COVID-19 vaccination and routine immunization uptake in the community. Their engagement in CAB meetings enabled them to share concerns, perspectives or misconceptions observed from their congregations, as well as identify effective means to address these with other CAB members. The involvement of faith leaders in the CAB, as well as other aspects of the intervention, facilitated unprecedented trust in vaccination in Mewat.

Along with being a platform to bring together the faith leaders with other members of the community, the CAB meetings were used as a means for our research team to connect with the community to obtain their thoughts on the project interventions. These monthly CAB meetings were recorded with along with extensive field notes maintained by the research staff to document these meetings. Two independent readers conducted rapid qualitative evaluation and thematic analysis of the data from each of the monthly CAB meetings to understand the impact of the intervention on the community.

## Results

The CIVIC project attempted to use a comprehensive and nuanced approach by involving faith leaders throughout the duration of the project, enabling them to play an active role in the intervention design. Not only did we encourage them to participate in advocacy efforts, we aimed to thoroughly understand their perceptions and provide them with a space to discuss concerns. As a result, we were able to gain their trust and earn their willingness to be advocates for vaccines. This comprehensive approach proved to be impactful in two diverse ways: first, through enhancing the community members' trust and acceptance in vaccines, and second, by supporting community health workers in their communications with caregivers.

Community members who expressed distrust in vaccines and were skeptical about the potential side effects felt reassured by seeing a faith leader they recognized and respected support vaccines. The video disseminated *via* social media was helpful in motivating the mothers, their husbands, and their families to understand the benefits vaccines offer for their health and for their child. Caregivers themselves also noted the positive impact of this video:

*If our Maulana is asking to get the vaccination and also saying that the vaccines are good, then I will definitely get my [pregnant] wife vaccinated. And I will also get the child, which is to be born, fully vaccinated. (Husband of pregnant woman)*

The faith leader videos also served as a helpful supplement for CHWs to improve their communication with caregivers regarding vaccines. Leveraging WhatsApp to share videos of faith leaders advocating for vaccination aided their communications with caregivers, inspiring willingness to receive vaccines for themselves and their children.

*The religious leader video has been a very useful aspect of the intervention. During talks with the caregiver, I have shown these videos while communicating about vaccination then they suddenly made a decision for [accepting a] vaccine. (Community Health Worker)*

## Discussion

As evidenced by our preliminary conversations with community members, faith leaders are valued members of the community members in Mewat, whose opinions are held in high regard.

Although previous projects have involved faith leaders, including The Pulse Polio Immunization program in India, these approaches were designed to involve the faith leaders in advocating for vaccines. All engagement activities began with the goal of understanding the “underlying reasons for the resistance” to the polio program, and, at this point, key faith leaders who could be involved in the polio program were identified. However, the program results were not encouraging, as faith leaders still felt a high degree of misapprehension about polio vaccines (6).

Our results show that it is essential to involve faith leaders in a sensitive, consistent, and long-term manner. Their comprehensive role in not only the advocacy efforts but also in the formative research, design and dissemination of interventions, and follow-up activities to improve vaccination proved to be a fruitful investment in our project and may be impactful in future work. Additionally„ the creation and involvement of faith leaders in the CAB meetings provides a long-term platform for community members and faith leaders to continue to meet and engage on vaccine and other health related issues beyond the duration of the project. These CAB meetings developing community accountability and ensuring long term sustainability of these interventions.

Globally, faith leaders have played a dynamic role in the COVID-19 response and have supported their communities to overcome vaccine hesitancy (24, 25). Even prior to the pandemic, faith leaders were recognized as capable of organizing and cultivating community participation in health issues (18). Amplifying their broad involvement by understanding their concerns, earning their trust, and then empowering them to use their voices to improve vaccine-seeking behaviors in communities has the potential to increase COVID-19 vaccine uptake and stimulate lasting trust in vaccines. In India, studies have shown that “hot spots” of negative or unclear information on COVID-19 vaccination can be addressed by involving friendly organizations who can clear strong disbeliefs (26). Faith leaders often gatekeepers of their communities and our results show that they can be powerful agents of change to facilitate vaccine confidence among hesitant communities.

Recognizing the unique position of faith leaders in communities, India's Ministry of Health and Family Welfare's COVID-19 Vaccine Communication strategy has highlighted their involvement as a key component of the vaccine advocacy strategy (27). This communication strategy from the Indian government recommends faith leader involvement in social mobilization activities and organizing advocacy events with faith leaders and faith-based institutions, complemented with written appeals and audio/video bytes for mass social media dissemination (27). However, to ensure long term sustainability, it is important for the interventions to be context specific and feasible with resources accessible to the community. Creating nuanced interventions that take into account community-specific hesitations, and work with faith leaders as vaccine advocates, is a critical grassroots-level strategy to improving vaccine acceptance across India.

Exploring which community influencers are most critical across different settings and employing “bottom-up” approaches to partner with communities to design interventions may help enhance trust and address inequities in a targeted manner (28, 29). The CAB brought together multiple stakeholders who play diverse roles in the community and were eager to share their thoughts and ideas for the improvement of their community's health and development. Toward the end of the project, the CAB felt that while progress had been made in terms of vaccination, they wanted to continue the meetings to discuss issues beyond vaccination to work toward improving broader issues in Mewat. Through their continued role in CAB, the faith leaders can bring the lens of religion, spirituality and faith to community discussions and decisions. This perspective is crucial to designing and implementing behavior change strategies that will resonate with the community and be sustained in the future (30).

In the past, faith leaders' involvement in health communication has led to favorable outcomes in tackling misinformation and reducing the spread of infectious diseases including polio, HIV and Ebola (4). Their involvement has proved to be crucial in global advocacy for polio vaccines, reducing stigma associated with HIV and changing religious practices associated with burials that were a source of Ebola exposure and infection. Working with individuals at all levels of community, particularly faith-based groups, has shown great value in several health emergencies (5). Tailoring interventions to meet specific needs of communities is crucial to optimizing long-term success and promoting equity. As faith leaders in certain closed-knit communities such as Mewat play a large role in influencing community health behaviors, we involved them in co-creating interventions specific to the needs of the community. We have shown how innovative ways of involving faith leaders to facilitate the success of health promotion programs, particularly in the context of COVID-19 vaccine uptake and recovering from disruptions in routine immunization services in low-coverage areas, bears potential for positive outcomes (4).

## Strengths and weaknesses of our approach

A unique strength of this project was that it was designed to be implemented completely virtually as a result of the COVID-19 pandemic. Despite the challenge of conducting community outreach in a rural setting without in-person meetings, performing project activities virtually enabled delivery of reliable vaccine messaging in a safe manner, adhering to movement restrictions and social distancing norms during the pandemic. This approach also allowed increased participation of community members and CHWs in the interventions, as they were able to join meetings from their homes without needing to travel long distances, limiting interference with their familial or professional responsibilities.

While our narratives in Mewat are descriptive of a small sample, our work suggests that the approach of involving faith leaders as champions for vaccines through dissemination of video messaging and involving them in decision making bodies was successful in promoting vaccine uptake and may be effective in similar areas of low vaccine uptake. However, a limitation of our work was voluntary response bias, as the study team could only engage with faith leaders who were willing to understand more about vaccines and engage in promotion activities. We were not able to access all the faith leaders across the community.

Lastly, the faith leaders involved in the project activities were all male. Going forward, exploring and enabling the involvement of women religious leaders in community decisions pertaining to health would be beneficial. Previous research has indicated that providing women with the opportunities to lead communities has been associated with higher educational attainment for girls, curbed negative attitudes toward female empowerment, and greater agency among women for decision making (31–33).

In this report, we have highlighted the process by which faith leaders' concerns about vaccines were understood and addressed, followed by their engagement in vaccine promotion activities. We have also described the positive influence of these activities. To gather a deeper understanding of the impact of these interventions, we plan to undertake a systematic evaluation of these approaches in future research.

By supporting and guiding their non-traditional leadership, along with innovative and advanced modes of message delivery, we have shown how faith leaders can facilitate long-lasting strategies to improve vaccine acceptance in their communities.

## Data availability statement

The raw data supporting the conclusions of this article will be made available by the authors, without undue reservation.

## Ethics statement

The study was reviewed by the Johns Hopkins Bloomberg School of Public Health (JHSPH) Institutional Review Board (IRB). Written informed consent for participation was not required for this study in accordance with the national legislation and the institutional requirements, however oral consent was taken.

## Author contributions

BD, RS, and ASh conceptualized and designed the project. BD, YQ, and BT designed data collection tools and facilitated interviews. BD, ASu, and PB analyzed and interpreted the study data. PB, RS, and BD drafted the initial manuscript. RS, ASu, ASh, and SC provided critical review of the manuscript. All authors reviewed the manuscript and approved the final manuscript as submitted.

## Funding

The CIVIC study was funded through the Sabin Vaccine Institute's Social and Behavioral Interventions for Vaccine Acceptance Small Grants Program under agreement number 050108-00.

## Conflict of interest

The authors declare that the research was conducted in the absence of any commercial or financial relationships that could be construed as a potential conflict of interest.

## Publisher's note

All claims expressed in this article are solely those of the authors and do not necessarily represent those of their affiliated organizations, or those of the publisher, the editors and the reviewers. Any product that may be evaluated in this article, or claim that may be made by its manufacturer, is not guaranteed or endorsed by the publisher.

## References

[1] Milstein G Palitsky R Cuevas A. The religion variable in community health promotion and illness prevention. J Prev Interv Community. (2020) 48:1–6. 10.1080/10852352.2019.1617519 31402789

[2] Ten Threats to Global Health in 2019. Available online at: https://www.who.int/news-room/spotlight/ten-threats-to-global-health-in-2019 (accessed May 18, 2022).

[3] Hatala A Pervaiz MC Handley R Vijayan T. Faith based dialogue can tackle vaccine hesitancy and build trust. BMJ. (2022) 376:o823 10.1136/bmj.o823 35346957

[4] Barmania S Reiss MJ. Health promotion perspectives on the COVID-19 pandemic: the importance of religion. Glob Health Promot. (2021) 28:15–22. 10.1177/1757975920972992 33228465 PMC8072799

[5] Obregón R Chitnis K Morry C Feek W Bates J Galway M . Achieving polio eradication: a review of health communication evidence and lessons learned in India and Pakistan. Bull World Heal Organ. (2009) 87:624–30. 10.2471/blt.08.060863 19705014 PMC2733260

[6] Eradicating Polio-Working With Religious Leaders to Enhance Community Ownership. Available online at: https://iple.unicef.in/files/ckuploads/files/Polio%20eradication%20in%20UP%20-%20Religious%20Leaders.pdf (accessed May 18, 2022).

[7] Bellatin A Hyder A Rao S Zhang PC McGahan AM. Overcoming vaccine deployment challenges among the hardest to reach: lessons from polio elimination in India. BMJ Glob Heal. (2021) 6:e005125. 10.1136/bmjgh-2021-005125 33906848 PMC8088252

[8] Deutsch N Singh P Singh V Curtis R Siddique AR. Legacy of polio—use of India's social mobilization network for strengthening of the universal immunization program in India. J Infect Dis. (2017) 216:S260–6. 10.1093/infdis/jix068 28838190 PMC5854010

[9] Chandani S Jani D Sahu PK Kataria U Suryawanshi S Khubchandani J . COVID-19 vaccination hesitancy in India: state of the nation and priorities for research. Brain Behav Immun Health. (2021) 18:100375. 10.1016/j.bbih.2021.100375 34693366 PMC8523306

[10] Sharma M Singh SK Sharma L Dwiwedi MK Agarwal D Gupta GK . Magnitude and causes of routine immunization disruptions during COVID-19 pandemic in developing countries. J Family Med Prim Care. (2021) 10:3991–7. 10.4103/jfmpc.jfmpc_1102_21 35136757 PMC8797101

[11] Shet A Dhaliwal B Banerjee P Carr K DeLuca A Britto C . COVID-19-related disruptions to routine vaccination services in India: a survey of pediatric providers. BMJ Paediatr Open. (2021) 5:e001060. 10.1136/bmjpo-2021-001060 34192200 PMC8047547

[12] Ministry of Health and Family Welfare District Fact Sheet Mewat Haryana. Available online at: http://rchiips.org/nfhs/NFHS-5_FCTS/HR/Mewat.pdf (accessed March 14, 2022).

[13] National Immunization Schedule (NIS) for Infants Children and Pregnant Women. Available online at: https://main.mohfw.gov.in/sites/default/files/245453521061489663873.pdf (accessed March 14, 2022).

[14] Ministry of Health and Family Welfare India Fact Sheet. Available online at: http://rchiips.org/nfhs/NFHS-5_FCTS/India.pdf (accessed March 14, 2022).

[15] Global Vaccine Action Plan: Monitoring Evaluation and Accountability. Secretariat Annual Report 2020. World Health Organization (2020).

[16] Dhaliwal BK Chandrashekhar R Rattani A Seth R Closser S Jain A . Community perceptions of vaccination among influential stakeholders: qualitative research in rural India. BMC Public Health. (2021) 21:2122. 10.1186/s12889-021-12188-4 34794415 PMC8600485

[17] Machingaidze S Wiysonge CS. Understanding COVID-19 vaccine hesitancy. Nat Med. (2021) 27:1338–9. 10.1038/s41591-021-01459-7 34272500

[18] Heward-Mills NL Atuhaire C Spoors C Pemunta NV Priebe G Cumber SN. The role of faith leaders in influencing health behavior: a qualitative exploration on the views of Black African Christians in leeds, United Kingdom. Pan Afr Med J. (2018) 30:199. 10.11604/pamj.2018.30.199.15656 30574218 PMC6294965

[19] Sabin Vaccine Institute. Community-led Strategies to Aid Vaccine Acceptance: Five Case Studies from the Global South. Washington, DC (2022). Available online at: https://www.vaccineacceptance.org/app/uploads/2022/03/Grant-Report_FINAL_v3.pdf (accessed May 18, 2022).

[20] Holeman I Kane D. Human-centered design for global health equity. Inf Technol Dev. (2019) 26:477–505. 10.1080/02681102.2019.1667289 32982007 PMC7484921

[21] Dhaliwal BK Singh S Sullivan L Banerjee P Seth R Sengupta P . Love, labor and loss on the frontlines: India's community health workers straddle life and the COVID-19 pandemic. J Glob Health. (2021) 11:03107. 10.7189/jogh.11.03107 35003708 PMC8719308

[22] Ballard M Bancroft E Nesbit J Johnson A Holeman I Foth J . Prioritizing the role of community health workers in the COVID-19 response. BMJ Glob Health. (2020) 5:e002550. 10.1136/bmjgh-2020-002550 32503889 PMC7298684

[23] India Covid-19: The Community Radio Station Fighting Fake News—BBC News. Available online at: https://www.bbc.com/news/av/world-asia-india-57635763 (accessed May 18, 2022).

[24] Wijesinghe MSD Ariyaratne VS Gunawardana BMI Rajapaksha RMNU Weerasinghe WMPC Gomez P . Role of religious leaders in COVID-19 prevention: a community-level prevention model in Sri Lanka. J Relig Health. (2022) 61:687–702. 10.1007/s10943-021-01463-8 34812996 PMC8609254

[25] Churches are Boosting Vaccination Rates in Eswatini. Gavi, The Vaccine Alliance. Available online at: https://www.gavi.org/vaccineswork/churches-are-boosting-vaccination-rates-eswatini (accessed January 25, 2022).

[26] Umakanthan S Patil S Subramaniam N Sharma R. COVID-19 vaccine hesitancy and resistance in India explored through a population-based longitudinal survey. Vaccines. (2021) 9:1064. 10.3390/vaccines9101064 34696172 PMC8537475

[27] COVID-19 Vaccine Communication Strategy 2 Gaj Ki Doori. Available online at: https://www.thehinducentre.com/resources/article33456011.ece/binary/Covid19%20Communication%20Strategy%202020.pdf (accessed March 14, 2022).

[28] Baum F. Cracking the nut of health equity: top down and bottom up pressure for action on the social determinants of health. Promot Educ. (2007) 14:90–5 10.1177/10253823070140022002 17665710

[29] A ‘Bottom-up’ Approach for COVID-19 Vaccines. VOX, CEPR Policy Portal. Available online at: https://voxeu.org/article/bottom-approach-covid-19-vaccines (accessed January 26, 2022).

[30] Kibongani Volet A Scavone C Catalán-Matamoros D Capuano A. Vaccine hesitancy among religious groups: reasons underlying this phenomenon and communication strategies to rebuild trust. Front Public Heal. (2022) 10:127. 10.3389/fpubh.2022.824560 35198525 PMC8858841

[31] Female Politicians Inspire Women In India To Pursue More Education MIT Study Finds. HuffPost. Available online at: https://www.huffpost.com/entry/mit-study-india-female-leaders-politicians-aspirations_n_1213998 (accessed May 18, 2022).

[32] Gangadharan L Jain T Maitra P Vecci J. Social identity and governance: the behavioral response to female leaders. Eur Econ Rev. (2016) 90:302–25. 10.1016/j.euroecorev.2016.01.003

[33] Jensen R. Do labor market opportunities affect young women's work and family decisions? Experimental evidence from India. Q J Econ. (2012) 127:753–92. 10.1093/qje/qjs002

